# Individually Tailored, Adaptive Intervention to Manage Gestational Weight Gain: Protocol for a Randomized Controlled Trial in Women With Overweight and Obesity

**DOI:** 10.2196/resprot.9220

**Published:** 2018-06-08

**Authors:** Danielle Symons Downs, Jennifer S Savage, Daniel E Rivera, Joshua M Smyth, Barbara J Rolls, Emily E Hohman, Katherine M McNitt, Allen R Kunselman, Christy Stetter, Abigail M Pauley, Krista S Leonard, Penghong Guo

**Affiliations:** ^1^ Exercise Psychology Laboratory Department of Kinesiology, College of Health and Human Development The Pennsylvania State University University Park, PA United States; ^2^ Department of Obstetrics and Gynecology College of Medicine The Pennsylvania State University Hershey, PA United States; ^3^ Center for Childhood Obesity Research Department of Nutritional Sciences, College of Health and Human Development The Pennsylvania State University University Park, PA United States; ^4^ Control Systems Engineering Laboratory School for Engineering of Matter, Transport, and Energy Arizona State University Tempe, AZ United States; ^5^ Department of Biobehavioral Health College of Health and Human Development The Pennsylvania State University University Park, PA United States; ^6^ Department of Nutritional Sciences College of Health and Human Development The Pennsylvania State University University Park, PA United States; ^7^ Department of Public Health Sciences College of Medicine The Pennsylvania State University Hershey, PA United States

**Keywords:** adaptive intervention, randomized controlled trial, mHealth, intervention study, overweight, obesity, pregnant women, exercise, nutrition science, gestational weight gain, body weight maintenance

## Abstract

**Background:**

High gestational weight gain is a major public health concern as it independently predicts adverse maternal and infant outcomes. Past interventions have had only limited success in effectively managing pregnancy weight gain, especially among women with overweight and obesity. Well-designed interventions are needed that take an individualized approach and target unique barriers to promote healthy weight gain.

**Objective:**

The primary aim of the study is to describe the study protocol for Healthy Mom Zone, an individually tailored, adaptive intervention for managing weight in pregnant women with overweight and obesity.

**Methods:**

The Healthy Mom Zone Intervention, based on theories of planned behavior and self-regulation and a model of energy balance, includes components (eg, education, self-monitoring, physical activity/healthy eating behaviors) that are adapted over the intervention (ie, increase in intensity) to better regulate weight gain. Decision rules inform when to adapt the intervention. In this randomized controlled trial, women are randomized to the intervention or standard care control group. The intervention is delivered from approximately 8-36 weeks gestation and includes step-ups in dosages (ie, Step-up 1 = education + physical activity + healthy eating active learning [cooking/recipes]; Step-up 2 = Step-up 1 + portion size, physical activity; Step-up 3 = Step-up 1 + 2 + grocery store feedback, physical activity); 5 maximum adaptations. Study measures are obtained at pre- and postintervention as well as daily (eg, weight), weekly (eg, energy intake/expenditure), and monthly (eg, psychological) over the study period. Analyses will include linear mixed-effects models, generalized estimating equations, and dynamical modeling to understand between-group and within-individual effects of the intervention on weight gain.

**Results:**

Recruitment of 31 pregnant women with overweight and obesity has occurred from January 2016 through July 2017. Baseline data have been collected for all participants. To date, 24 participants have completed the intervention and postintervention follow-up assessments, 3 are currently in progress, 1 dropped out, and 3 women had early miscarriages and are no longer active in the study. Of the 24 participants, 13 women have completed the intervention to date, of which 1 (8%, 1/13) received only the baseline intervention, 3 (23%, 3/13) received baseline + step-up 1, 6 (46%, 6/13) received baseline + step-up 1 + step-up 2, and 3 (23%, 3/13) received baseline + step-up 1 + step-up 2 +step-up 3. Data analysis is still ongoing through spring 2018.

**Conclusions:**

This is one of the first intervention studies to use an individually tailored, adaptive design to manage weight gain in pregnancy. Results from this study will be useful in designing a larger randomized trial to examine efficacy of this intervention and developing strategies for clinical application.

**Registered Report Identifier:**

RR1-10.2196/9220

## Introduction

### Background

Maternal weight gain in pregnancy is necessary for healthy fetal development [1]. The Institute of Medicine [1] recommends that appropriate weight gain for normal weight women is 25-35 pounds, whereas weight gain for overweight (15-25 pounds) and obese (11-20 pounds) women is less; any weight above these upper guidelines is considered excessive or high. High gestational weight gain (GWG) is strongly related to and independently predicts adverse pregnancy outcomes (eg, preterm delivery, gestational diabetes, and pre-eclampsia), greater adiposity, postpartum weight retention, and long-term obesity and cardiovascular disease [1,2]. Maternal obesity and high GWG also elevate infant risks such as macrosomia and early onset of obesity and diabetes [1]. As GWG is a modifiable factor that can be targeted to reduce adverse risks, managing GWG is critical for the mother, and can influence the etiology of obesity for offspring at a crucial time in the life cycle. However, over 50% of women enter pregnancy as already overweight (body mass index, BMI, 25-29.9 kg/m^2^) or obese (BMI ≥30 kg/m^2^) [3], and nearly 60% of women with overweight/obesity gain more weight in pregnancy than that recommended by the Institute of Medicine [1], which suggests they have particular challenges with managing their weight.

Past randomized intervention trials have shown that behavioral interventions can effectively manage weight in pregnant women [4-7] when they “mirror” effective programs in nonpregnant women such as using calorie goals, frequent contact and weight and dietary intake monitoring, and promoting healthy eating and exercise behaviors [8-10]. However, the effectiveness of these interventions has mainly been demonstrated in normal weight women. The results of studies including women with overweight and obesity are mixed. Several studies found little to no effect on GWG [5,6,11-13], whereas other interventions observed some effects [14,15] but were based on older guidelines [16] that were less restrictive than the current guidelines [1]. One study using current guidelines found a significant reduction in GWG (23 pounds) among women with obesity participating in a lifestyle intervention group compared with the 30 pound gain in the control group [17]. Both groups, however, exceeded the Institute of Medicine GWG guidelines [1]. In short, women with overweight and obesity have unique challenges with managing weight that may likely require a more intensive and tailored approach than what is traditionally delivered in usual prenatal care or has been tested in past interventions.

One strategy that has been used to facilitate behavior change in other areas (eg, mental health issues, child behaviors, weight loss) [18-20] is an adaptive intervention (also known as a dynamic treatment regime or adaptive treatment strategy) [21,22]. In an adaptive intervention, a sequence of intervention strategies or dosages is tailored to an individual’s needs (similar to clinical practice) as opposed to a “one size fits all” intervention approach [23]. Adaptive interventions include critical decision points (eg, which intervention to start with, when and how to measure signs of response or nonresponse), intervention components (eg, set of intervention options at each critical decision point), tailoring variables (eg, variables expected to impact the effect of the component), and decision rules (eg, links tailoring variables to the intervention components) [24]. Moreover, principles of control systems engineering can be applied to an intervention context by using decision algorithms (controllers) to regulate a dynamical system to optimize intervention outcomes [25]. For example, control systems engineering can be used to construct and estimate a dynamical model that considers how changes in an outcome (eg, weight) are related to planned and self-regulatory behaviors (eg, dietary intake, physical activity) over time. An intervention that helps each pregnant woman with overweight and obesity to control GWG on a weekly basis and adapts to her unique needs over the course of pregnancy may be a promising strategy for managing weight in pregnancy. However, we are not aware of prior research that has used this intervention approach to manage weight gain in pregnancy.

### Objectives

The primary aim of this publication is to describe the study protocol for Healthy Mom Zone, an individually tailored, adaptive intervention for managing weight in pregnant women with overweight and obesity. We will explain the conceptual framework, theoretical components, intervention dosages, and decision rules for how and when to adapt the intervention. We then describe the intensive longitudinal data collection procedures and the methods for conducting poststudy participant interviews to examine program compliance, dosage exposure, and participant responsiveness. We will also explain the plan to establish initial validation of the intervention by examining impacts of the program on GWG (ie, average weekly GWG of intervention vs control group; meeting individual GWG goals based on prepregnancy weight status), energy intake, physical activity, planned self-regulatory behaviors, and related maternal health outcomes. This work aims to use principles of control systems engineering to optimize the intervention for future application; in other words, to inform how the intervention can effectively and efficiently manage GWG in pregnant women with overweight and obesity.

## Methods

### Ethics Approval and Consent to Participate

This study has been approved by the Pennsylvania State University Institutional Review Board (Study #00000122) and Clinical Research Center (#319). All legal and data protection issues are discussed with the responsible authorities, and all participants are required to provide active informed consent.

### Overview of Healthy Mom Zone Study Design

**Figure 1:**
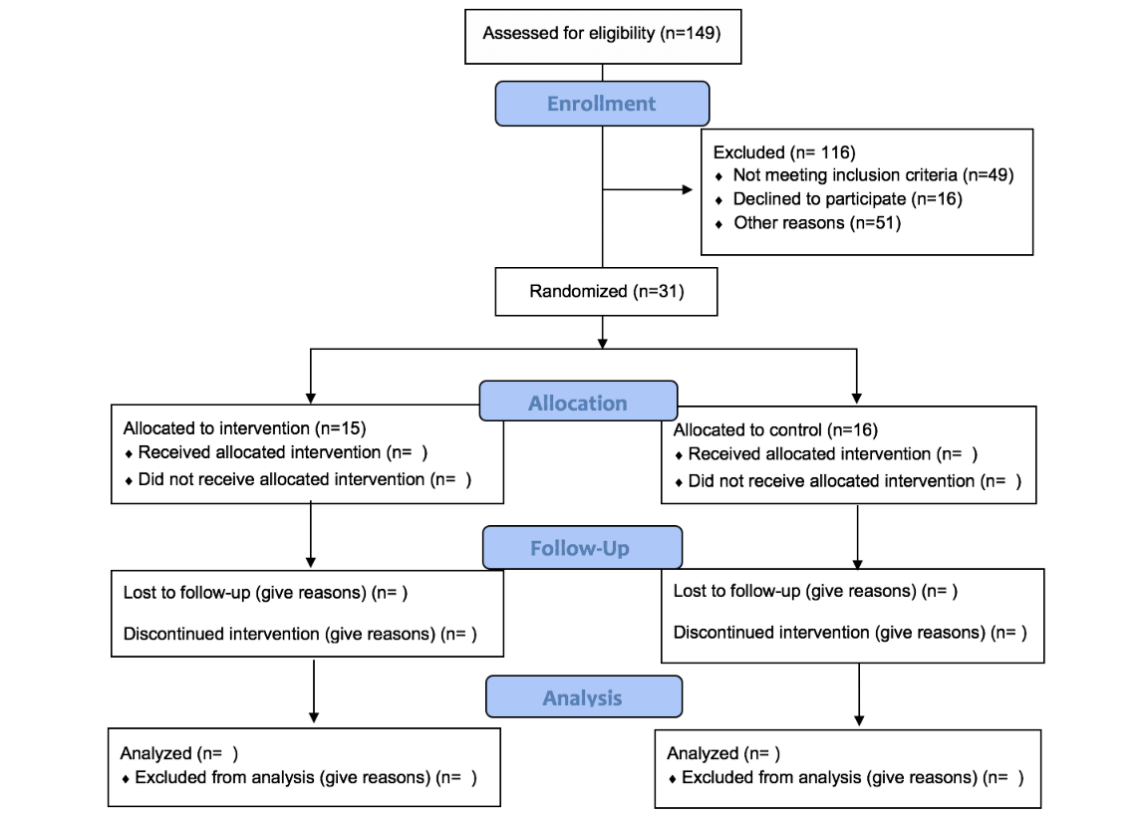
Flow of participants through Healthy Mom Zone Intervention (still underway) as per Consolidated Standards for Reporting Trials guidelines. Participants allocated to intervention group could be assigned to various step-ups throughout the intervention time period. Step-ups will vary by individual and time-point of intervention and gestation. Incomplete cells are due to ongoing data collection.

The intervention is currently being tested in a longitudinal study design among a cohort of pregnant women with overweight and obesity randomized to either the intervention or control group from approximately 8-36 weeks gestation. Figure 1 summarizes the flow of participants through the study using the Consolidated Standards of Reporting Trials template [26]. Study measures are obtained at baseline, throughout the course of the intervention (eg, daily, weekly, and monthly), and at follow up (see Table 1). GWG is evaluated weekly and decision rules are used in 3-4 week cycles to decide when and how the intervention may be adapted for an individual. The decision to evaluate GWG in 3-4 week cycles was based on the following: (1) clinical insight and judgment (eg, prenatal care visits for most women occur in 3-4 week cycles, so we used the same cycle for our GWG evaluations), (2) reducing participant burden (eg, the 3-4 week cycle allowed women to adjust to the dosages before adaptations are made), and (3) being proactive about adjusting the intervention dosage in real-time before a woman’s weight exceeds her goal. In addition, mHealth tools are used to allow participants to self-monitor behaviors (ie, weight, physical activity, dietary intake). The study staff can review the data collected from the monitors and provide the participants with real-time feedback to facilitate compliance (eg, contact participants when the devices are not working or not being used according to the study protocol). All participants are compensated for their time spent on completing the study measures (receive gift cards and cash for pre- and postmeasures); women participating in the intervention do not have to return their Wi-Fi weight and food scales. We will use the data collected in this study and dynamical modeling from control systems engineering (system identification) to express progression of GWG over time. We will identify a customized intervention plan for each woman based on her energy intake, physical activity, planned and self-regulatory behaviors, and the extent to which she is meeting GWG goals over pregnancy. This will lead to final intervention modifications and result in an individually tailored, adaptive and optimized intervention, of which we will test efficacy in a future randomized controlled trial.

**Table 1:** Healthy Mom Zone measurement protocol. X designates when the measurement had taken place. All participants completed this measurement protocol.

Variable	Assessment	Time point						
		Pr^a^	Po^b^	D^c^	W^d^	BiW^e^	M^f^	Pp^g^
Weight and weight gain	High Precision Stand-On Adult Scale	X	X					X
	FitBit Aria Wi-Fi Smart Scale			X				X
Height	Stadiometer	X						
Serum blood	Macronutrients	X	X					
Adiposity	Air-Displacement Plethysmography (BODPOD) Body Composition	X	X					X
Healthy eating behaviors	MyFitnessPal Phone App				X			
	3-Factor Eating Questionnaire	X	X				X	
	Remote food photography		X					X
Physical activity behaviors	Actigraph (time spend in moderate-intensity activity)	X	X				X	
	Jawbone Wrist-Worn Activity Monitor	X	X	X				X
	Physical activity log (mode and min activity)							X
	Leisure-Time Exercise Questionnaire	X	X				X	
Weight attitude	Palmer attitudes toward weight in pregnancy	X	X				X	
Healthy eating and exercise motivational determinants	Attitude, subjective norm, perceived control, intention	X	X				X	
	Healthy eating and exercise beliefs	X	X		X			
Self-regulation	Self-regulation index	X	X			X		
	Self-Regulation Questionnaire (self-regulatory function)	X	X			X		
	Self-control scale	X	X				X	
Sociodemographic	Health history Questionnaire	X						
	Pregnancy complications	X			X			
Self-efficacy	Sui Healthy Eating and Physical Activity Self-Efficacy Scales	X	X				X	
	Clark Weight Efficacy Scale	X	X				X	
Sleep behaviors	Pittsburgh Sleep Quality Index	X	X				X	
	Jawbone Wrist-Worn Activity Monitor	X	X	X				X
Psychosocial	Center for Epidemiological Studies Depression Scale	X	X				X	
	State Trait Anxiety Inventory	X	X			X	X	
	Body Areas Satisfaction Scale	X	X				X	
	Perceived Stress Scale	X	X		X			
	Pregnancy Figure Rating Scale	X	X				X	
	Rothbart Adult Temperament Scale	X						
Stress	Urine cortisol collection	X	X		X			X
Metabolism	Breezing device	X	X		X			X
Fetal Growth	Standard fetal ultrasound scans						X	

^a^Pr: preintervention.

^b^Po: postintervention.

^c^D: daily.

^d^W: weekly.

^e^BiW: biweekly.

^f^M: monthly.

^g^Pp: postpartum.

### Participants and Sample Size

The target sample for this intervention is 30 pregnant women with overweight and obesity (BMI range 25-45 kg/m^2^; >40 kg/m^2^ with physician consultation); singleton pregnancy >8 weeks gestation; English-speaking; residing in or near Centre County, Pennsylvania; and with physician consent to participate. On the basis of our past research [27,28], this group compromises >85% of live births in Central Pennsylvania. Exclusion criteria are multiple gestation, diabetes at study entry, not overweight or obese, severe allergies or dietary restrictions, contraindications to prenatal physical activity [29], and not residing in area for duration of the study. The primary outcome of our study is GWG. Julious [30] effectively argues a sample size of 12 per group is adequate to assess feasibility and obtain sufficient precision of the mean and variance to perform a formal sample size and power calculation for a future larger randomized control trial with GWG as the primary outcome. With 24 participants (12 per group), we will have 80% statistical power to detect a standardized effect size (difference between 2 group means divided by SD) for GWG of 1.2 using a 2-sided test with significance level of *P*=.05. Factoring in participant dropout (conservative estimate of 25% based on our pilot work [31,32] and past research [5]), we will recruit 30 participants (15 intervention, 15 control).

### Recruitment, Assignment, and Allocation

Women are recruited using on-site clinic, community-based, and Web-based strategies. At the clinic, nurses identify potential eligible women and refer them to a study staff member who is ready in the waiting room to speak with them about the study and, if interested, screen them for eligibility. Community-based recruitment includes hanging study flyers at local businesses, churches, campus, and community centers. Web-based recruitment occurs through the study website [33] and Facebook ads. Interested women call a toll-free number. A project staff member explains the study, obtains verbal assent to ask questions, and determines eligibility based on the inclusion criteria described above. Pregnant women with overweight and obesity are recruited between 8 and 12 weeks gestation and randomized to the intervention or control condition until about 36 weeks gestation. The study biostatisticians developed a randomization scheme using variable size, random permuted blocks to ensure the number of participants in each group is balanced after each set of *B* randomized participants, where *B* is the block size. Randomization to intervention (n=15) or control (n=15) groups uses 1:1 allocation; participants are entered consecutively. When a woman is eligible and informed consent is signed, a staff member requests randomization by a unique participant identification number.

### Conceptual Framework

**Figure 2:**
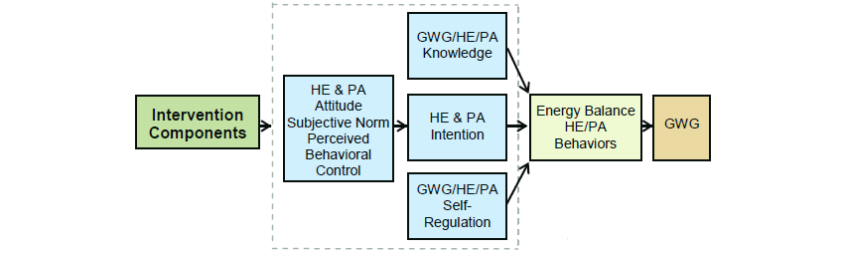
Conceptual framework for Healthy Mom Zone Intervention. HE: healthy eating; PA: physical activity, GWG: gestational weight gain.

We used principles from the Theory of Planned Behavior [34] and self-regulation [35] for the conceptual framework of the Healthy Mom Zone Intervention (Figure 2).

**Figure 3:**
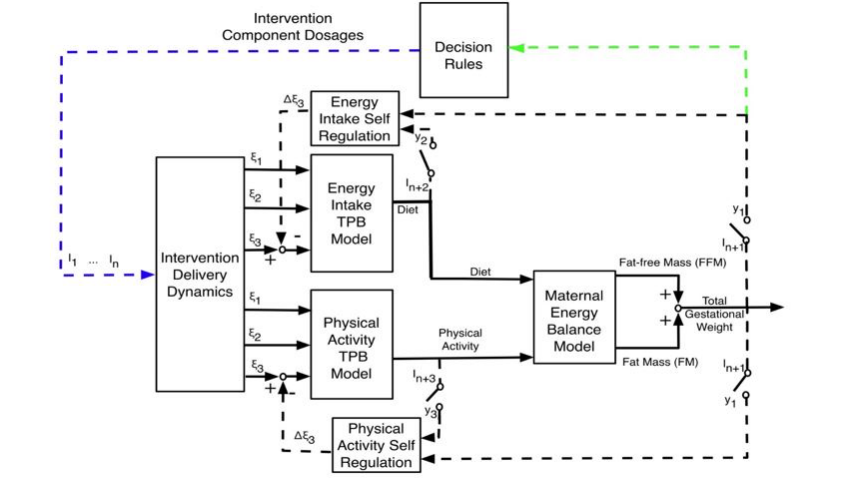
Energy balance model underlying the Healthy Mom Zone Intervention. TPB: Theory of Planned Behavior; I_1_…I_n_: Intervention components; i: exogenous variables that serve as inputs for behavioral models; y_i_: system outputs; ξ_1_: Behavioral belief × evaluation of outcome; ξ_2_: Normative belief × motivation to comply; ξ_3_: Control belief × power of control belief; I_1_: Healthy Eating Education; I_2_: Healthy Eating Weekly Plan; I_3_: Healthy Eating Active Learning; I_4_: Goal Setting; I_5_: Physical Activity Education; I_6_: Physical Activity Weekly Plan; I_7_: Physical Activity Session; I_8_: Daily Weight Scale; I_9_: Dietary Record; I_10_: PA monitor output. Black solid line shows input/output signals between models; Black dashed line shows self-regulation feedback loop; Blue dashed line shows intervention dosages which indicate how and when the intervention is adapted; Green dashed line shows tailoring variables that inform whether the intervention is adapted; Circle dashed line shows regular measurement of important outcomes (self-regulation intervention).

The Theory of Planned Behavior assumes a person is motivated for a behavior (eg, managing GWG) when she has a positive attitude, believes that significant others want her to do the behavior (subjective norm), and has the perceived ability to do the behavior (perceived behavioral control) [34]. Self-regulation assumes that behavior is goal-directed and regulated by feedback control processes [35]. The participant engages in activities in which she can succeed, and this confidence in performance success can influence her perceived behavioral control. Thus, self-monitoring is a core strategy of behavior modification and a key aspect of the dynamical systems model to manage GWG in this study. The overall simulation model for GWG is depicted in Figure 3 and includes the following: (1) a 2-compartment energy balance model predicting changes in body mass as a result of energy intake and physical activity, (2) 2 Theory of Planned Behavior models describing how energy intake and physical activity are affected by behavioral variables, (3) a program delivery module relating magnitude and duration of components to inflows of the Theory of Planned Behavior models, and (4) 2 self-regulation units modeling how success expectancies in the intervention influence one’s goal achievement motivation [36,37]. This model serves a vital role in evaluating the decision rules in the individually tailored intervention and in applying advanced strategies to produce decision frameworks for making program adaptations.

### Healthy Mom Zone Intervention Components

**Figure 4:**
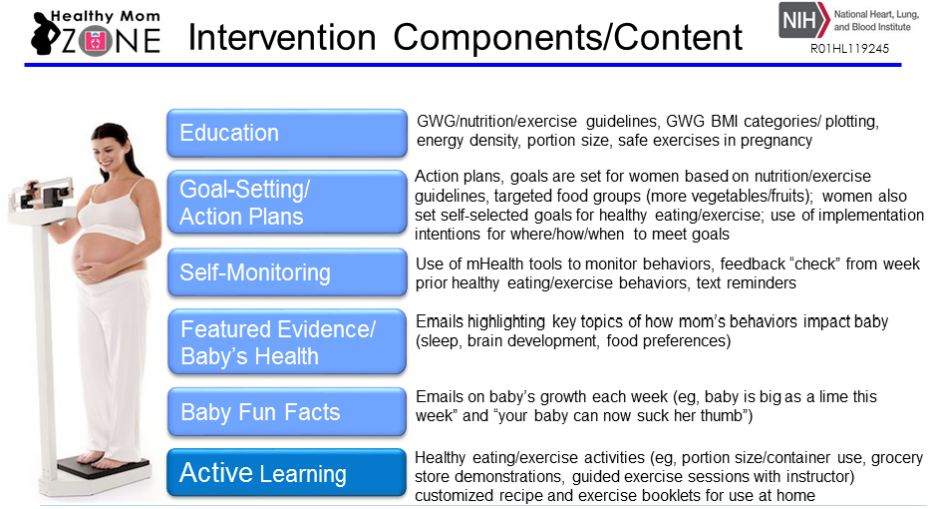
Healthy Mom Zone Intervention components. Components in light blue are in baseline intervention and delivered throughout the duration of the intervention. Active Learning component is adapted depending on decision rules and gestational weight gain (GWG) evaluations. BMI: body mass index.

The intervention components (see Figure 4) are informed by past research and our own pilot data [5-10,31,32,38,39]. Evidence from model lifestyle interventions [8-10,38], GWG interventions [5-7], and our research on promoting healthy behaviors [39-41] shows education, goal-setting and action planning, and self-monitoring can effectively manage weight. Our past studies have shown that when people are taught how to set appropriate plans and goals, self-monitor, and manage their time, they are more likely to achieve their goals and see positive behavioral outcomes (eg, engage in healthy eating and exercise to manage weight) [39,42-44]. Furthermore, convincing evidence from past research [39,44,45-49] shows that healthy eating and physical activity active learning (eg, active participation in strategies to reduce energy density such as food preparation and planning, portion size control, increasing intake of fruits and vegetables, meeting physical activity goals, and engaging in guided exercise sessions) are effective for lowering energy intake and managing body weight. We also learned from our pilot study [50] that women wanted to know more about how the target intervention behaviors (eg, weight, physical activity, dietary intake) were related to their baby’s growth, so we developed brief modules to inform women about the following: (1) current research studies (Featured Evidence and Baby’s Health) and (2) unique aspects of their baby’s growth (Baby Fun Facts); content is delivered weekly in this study via email.

### Standard of Care Control Condition

All women in the study receive standard prenatal care (eg, regular visits and prenatal counseling with health care provider; provider is not informed by the research team of randomization assignment). The women randomly assigned to the control condition also complete the same measurement protocol as the intervention participants, which includes daily, weekly, and monthly assessments (see Table 1).

### Healthy Mom Zone Intervention Description

Women randomized to the intervention group receive the baseline dosage, which includes for all women the intervention components (described above) of education, goal-setting and action plans, featured evidence on baby’s health, baby fun facts, and self-monitoring. Participants meet weekly with a study dietitian and are given customized calorie goals and a booklet developed for this study that contain customized healthy eating plans, recipes, and tailored meals to meet calorie goals. They are also given a physical activity booklet with customized and safe pregnancy-related exercises (see Multimedia Appendix 1). A study staff member reviews each woman’s weight, physical activity, and dietary intake via real-time data collection procedures and generates a report for the study dietitian to review with the participant at the next intervention session.

The dietitian plots weekly GWG using individualized charts that illustrate GWG within the context of the Institute of Medicine guidelines and discusses strategies to overcome barriers to managing weight and engaging in healthy eating and physical activity behaviors. Women in the intervention also complete a 15-min postintervention exit interview at their post assessment to provide their feedback and perspective on multiple aspects of their intervention experience (eg, likes and dislikes about using the mHealth tools, adaptive dosages, active learning activities, completing study measures). Intervention sessions are randomly audio-recorded and coded for fidelity.

### Study Setting and Procedures

All participants, regardless of intervention assignment, complete the same study measurement protocol. The study setting includes onsite visits at the Penn State Clinical Research Center (University Park campus) and at-home participation. Interested and eligible women complete a preintervention assessment at the Penn State Clinical Research Center after the study is described and written consent is obtained. A study clinician assesses height, weight, blood pressure, and conducts a physical exam to identify health symptoms that may preclude study participation [29]. Women complete self-reported measures of their healthy eating/physical activity behaviors, knowledge, planned and self-regulatory behaviors, motivational determinants, demographics, and dietary intake using onsite paper and pencil and Web-based data capture software (Research Electronic Data Capture) [51].

Women then complete an air displacement plethysmography body composition assessment following a standardized protocol that takes approximately 30 min. Participants complete these same measures again at the postassessment at approximately 36 weeks gestation. In addition, during the course of the study all women are given a Wi-Fi scale for wireless uploading of daily weight, food scale to monitor daily intake, and log to record food and beverages (and amounts) consumed, and 2 monitors (wrist-worn activity monitor worn daily; waist-worn accelerometer worn in 2-week cycles) to track physical activity. Participants are asked to use these mHealth devices continuously over the course of the entire study. Women track dietary intake on 2 weekdays and 1 weekend using a mobile phone app. Measures are obtained at baseline, during the intervention (daily, weekly, and monthly), and postintervention. All participants are compensated up to US $420 in check or gift card for the pre and post, weekly, and monthly assessments over the study period. In addition, the intervention participants get to keep their Wi-Fi weight and food scales at the end of the study (control participants return them), and they receive a US $20 check if they attend 90% (25/28) or more of the intervention sessions.

### Decision Rules and Adapting the Intervention Dosage

A set of decision rules were developed to evaluate GWG that are based on the Institute of Medicine GWG guidelines (ie, overweight=15-25 pounds; obese=11-20 pounds) [1], our own research [36,37,50], and clinical insight that inform when and how to adapt the components. A computerized applet is used to plot women’s weight against individual weight gain trajectories with lower and upper bounds for the GWG guidelines [52,53]; GWG is evaluated weekly and the values are compared with average weekly weight ranges for overweight (eg, 0.5-0.7 pounds) and obese (eg, 0.4-0.6 pounds) women [1]; these weekly weights are then evaluated in 3-4 week cycles to determine if and when the intervention dosage should be adapted (see Multimedia Appendix 2). The rationale for adapting the dosages is based on each woman’s self-regulatory abilities to manage GWG. For example, if the woman is within her GWG goal (ie, weekly weight gain ranges according to the Institute of Medicine guidelines) [1], she will continue to receive the same level of the intervention for the next cycle as she is adequately self-regulating her weight with the amount of intervention dosage that she’s receiving. However, if she is exceeding her GWG goal, the intervention is adapted (ie, dosage is stepped up). That is, she continues to receive the baseline intervention, but then she receives additional active learning components (eg, step-up 1 with cooking and grocery store demonstrations; onsite exercise session led by an instructor) to help better self-regulate her weight. If she exceeds her GWG goal at the next evaluation cycle, the intervention will be adapted again (ie, baseline + step-up 1 + step-up 2) with other active learning components (eg, portion size and containers; second onsite exercise session with instructor) added to provide more intensive intervention assistance and supportive control to better manage GWG. This evaluation process continues over the course of her pregnancy until the end of the intervention (ie, around 36 weeks gestation) and/or a maximum of 5 possible dosage increases (see Multimedia Appendix 3). The sequencing of the adaptive dosages was determined during pilot testing by examining combinations of active learning strategies with good user acceptability, and we identified the maximum number of dosage increases that resulted in “too much intervention burden” and led to participant dropout (ie, more than 5 adaptations) [50]. If a participant is under her GWG goal [1] and/or any safety concerns emerge (eg, complication such as anemia, fetal issue), we consult with the study obstetrician who makes recommendations on if and/or how intervention dosage changes should be made.

### Outcome Measures

The measurement protocol is presented above in Table 1. The primary outcome measure is GWG. Weight and GWG are assessed pre- and postintervention at the Penn State Clinical Research Center using standardized procedures (eg, measured in duplicate to nearest kilogram using a high-precision stand-on adult scale; wearing undergarments and gown) and daily at home using the Wi-Fi wireless scale. Secondary outcomes include adiposity (body composition), healthy eating behaviors, physical activity behaviors, knowledge, motivational determinants, self-regulation, psychological well-being, sleep, serum blood for macronutrients, stress (cortisol), metabolism, and fetal growth.

### Data Collection

Data on the primary and secondary outcome variables above are collected in addition to participant sociodemographic characteristics (eg, age, BMI status) to understand the influence of potential moderators on the primary and secondary outcome variables. Standardized procedures were developed for data collection, recording of errors, and a comprehensive data quality assurance program to ensure complete, accurate, and valid data while limiting variability in data recording. Study data are managed using a secure Web app that provides user-friendly Web-based case reports, real-time data entry validation audit trails, and a deidentified data export mechanism to commonly used statistical packages.

### Data Analysis

To establish initial validation of the intervention, contrasts constructed from linear mixed-effects models [54] will be used to assess differences between the intervention and control groups with respect to changes in continuous outcomes over time (from pre- to postintervention): changes in primary (GWG, energy intake, physical activity, planned/self-regulatory behaviors) and secondary outcomes (serum blood biomarkers, adiposity, knowledge). Linear mixed-effects models are an extension of ordinary regression models that account for within-subject variability inherent in longitudinal studies. Generalized estimating equations [55] with a logit link will be used to assess differences between the intervention and control groups with respect to dichotomous outcomes over time. If deemed necessary, confounding variables (eg, prepregnancy weight, age, obstetric complications) will be included as covariates. With respect to the participants meeting individual GWG goals based on prepregnancy BMI status [1], we will look at each woman and determine whether she met her GWG goal. We will code participants as “0” for GWG within goal, +1 for GWG over goal, and −1 for GWG under goal. A chi-square test will be used to assess differences between the intervention and control groups on this 3-level ordinal variable (ie, −1,0,1) assessing individual GWG goals. All hypothesis tests will be 2-sided, and analyses will be performed using SAS software version 9.4 (SAS Institute Inc., Cary, NC) or R software (R Foundation for Statistical Computing, Vienna, Austria).

Modeling development and simulation are done in Matlab with SIMULINK (The MathWorks, Inc., Natick, MA). Functional data analysis modeling for time-varying modeling will rely on SAS. Weight predictions based on a first-principles energy balance model can be performed using measurements of a participant’s energy expenditure (physical activity and resting metabolic rate) and energy intake. Alternatively, energy intake can be estimated from the energy balance model using the measured weight gain and physical activity, which can then be compared with self-reported data [56,57]; this information can be used by dieticians to provide informative health guidance. Intensive measurements of the Theory of Planned Behavior constructs are used to build participant-based dynamical models using semiphysical model identification techniques. The integration of these behavioral models with energy balance models can be used to evaluate and implement decision policies based on control systems engineering for an intensively adaptive intervention, specifically Hybrid Model Predictive Control [37,58].

## Results

The targeted sample of pregnant women with overweight and obesity has been successfully recruited between January 13, 2016, and July 1, 2017. Baseline data have been collected for all participants. To date, 24 participants have completed the study and postintervention follow-up assessments, 3 are currently in progress, 1 dropped out, and 3 women had early miscarriages and are no longer active in the study.

**Figure 5:**
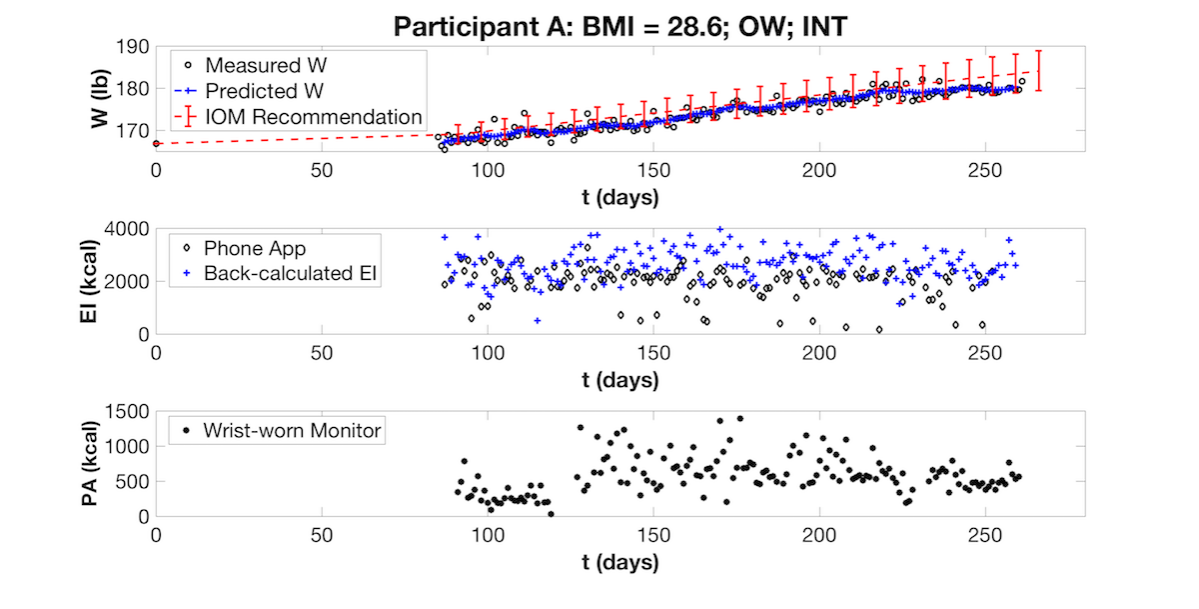
Preliminary visualization of a Healthy Mom Zone Intervention participant’s gestational weight gain data. The intervention participant’s measured weight is plotted against her predicted weight (based on the computerized applet) and the Institute of Medicine upper and lower ranges for recommended weight gain for a woman who is OW. Her EI (measured with phone app and estimated with a back-calculation formula) and PA (measured with wrist-worn activity monitor) are also plotted. BMI: body mass index; OW: overweight; INT: intervention participant; W: weight; EI: energy intake; PA: physical activity; kcal: kilocalories.

Of the 24 participants, 13 women have completed the intervention to date, of which 1 (8%, 1/13) received only the baseline intervention, 3 (23%, 3/13) received baseline + step-up 1, 6 (46.1%, 6/13) received baseline + step-up 1 + step-up 2, and 3 (23%, 3/13) received baseline + step-up 1 + step-up 2 + step-up 3.

Preliminary results are presented in Figure 5 for 1 participant who is overweight and was randomized to the intervention group (from gestational age day 85 through day 261). As shown in Figure 5, her actual weight remained within that recommended by the Institute of Medicine [1], whereas under-reporting is observed in her self-reported energy intake compared with a back-calculation method based on our maternal energy balance model ([56]; see reference for more detailed explanation of the back-calculation method). Using the back-calculated energy intake for weight prediction, the model-predicted weight follows closely to her measured weight. Data analysis is still ongoing through spring 2018.

## Discussion

### Principal Findings

To the best of our knowledge, this is the first study that is testing the feasibility of an individually tailored, adaptive intervention to manage GWG in pregnant women with overweight and obesity. This intervention, Healthy Mom Zone, aims to improve upon past interventions by addressing gaps in the literature, specifically among pregnant women with overweight and obesity, by providing a more individually tailored and adaptive approach to effectively manage weight gain during pregnancy. Although this intervention was deliberately intensive to understand if this approach can effectively manage GWG, future plans for this line of research will explore how the Healthy Mom Zone Intervention can be further adapted to women’s individual needs.

For example, we may learn from the study findings that some women can effectively manage their GWG with less intervention, and therefore, we can step down the intervention for them, whereas other women need the step up to keep their weight within their goals. This approach will also increase the clinical application and utility of the intervention.

### Strengths and Limitations

There are several positive features of this study. This study has strong public health relevance and clinical significance for the future management of GWG. We have also incorporated several innovative aspects into the study, including the use of (1) novel decision rules to choose when and how to adapt the intervention, (2) mHealth tools for self-monitoring of behaviors and real-time data collection to provide feedback to the participants, and (3) a unique computerized applet to generate individualized weight gain trajectories for comparing actual weight to the Institute of Medicine guidelines. In addition, customized eating and physical activity plans developed for this study aid in reducing participant barriers to engaging in healthy behaviors during pregnancy. Moreover, the intensive longitudinal data collection protocol allows for a myriad of systems approaches in support of implementing and evaluating intensively adaptive interventions [25,56-58]. Intensive data collection enables the application of system identification and state estimation approaches from engineering that, in turn, build comprehensive dynamical models for GWG used in adaptive intervention optimization. These methods can be used to estimate and correct energy intake over time, despite some missing data.

There are also some limitations of this research. The small sample size, although adequate for examination of the primary outcome (GWG), precludes the ability to make assumptions at a population level. Moreover, the target population is a homogenous sample of women from largely rural and suburban areas in Central Pennsylvania, thus limiting the extension of the study findings to more culturally diverse and urban populations of pregnant women with overweight and obesity. We do plan to understand the application of Healthy Mom Zone to a more diverse sample of pregnant women in the future, including those who are normal weight, more culturally diverse, and reside in varied communities across the United States.

### Conclusions

The data and knowledge obtained from this innovative intervention will be valuable for informing future studies aiming to manage GWG in pregnancy. We aim to learn from the study findings how to further adapt the intervention (eg, step up or down) to meet women’s needs and customize the intervention based on women’s individual characteristics. For example, we may learn that certain individual characteristics (eg, higher perceived behavioral control, lower stress) help women to self-regulate their weight better, and therefore, we can expand the intervention to better target these factors at the start of a pregnancy. The insight gained from this study as well as independent developments in mHealth tools and their use in clinical practice (eg, links to electronic records and provider communication) will also help to disseminate this individually tailored, adaptive intervention to effectively manage GWG in clinical practice—so that all pregnant women can be targeted to ultimately improve maternal and infant health outcomes and impact the etiology of obesity at a critical time in the life cycle.

## Appendix

Multimedia Appendix 1 Study booklets with customized healthy eating and physical activity material.

Multimedia Appendix 2 Illustration of the decision rule for GWG evaluation and adapting dosages.

Multimedia Appendix 3 Illustration of Healthy Mom Zone Intervention adaptive intervention design.

Multimedia Appendix 4 CONSORT-EHEALTH checklist (V 1.6.1).

## Abbreviations

BMI: body mass index
EI: energy intake
GWG: gestational weight gain
INT: intervention participant
kcal: kilocalories
PA: physical activity
TPB: Theory of Planned Behavior
