# Supplementary material for: Individually Tailored, Adaptive Intervention to Manage Gestational Weight Gain: Protocol for a Randomized Controlled Trial in Women With Overweight and Obesity
Source: JMIR Res Protoc. 2018 Jun 8;7(6):e150. doi: 10.2196/resprot.9220 (PMC6015270; doi:10.2196/resprot.9220)

IRB# CATS00003752
PENNSTATE

Healthy Mom ZONE

Fast, Easy & Healthy
Breakfast Recipes

Breakfast Sandwich Builder

Makes: 1 serving

All of these breakfast sandwiches can be made ahead and frozen individually for a perfect quick and easy breakfast! Pick your favorite combination and assemble into a sandwich. Individually wrap each sandwich in plastic wrap and transfer them into a gallon sized plastic bag for storage in the freezer. When you're ready to eat your sandwich in the morning, simply take one out of the freezer, unwrap it and microwave until desired temperature (30 seconds-1 minute). Then top with your favorite veggie and extra options!

Here are some examples, but the possibilities are endless!

BREAD	EGG	PROTEIN	VEGETABLE *ADD WHEN READY TO EAT	EXTRAS *ADD WHEN READY TO EAT
1 bagel thin	Hard boiled egg, sliced	Ham	Slice of tomato	Thin slice of low fat cheese
1 whole wheat English muffin	Scrambled	2 slices of Turkey Bacon	Mushroom and spinach (scrambled with egg)	Sprinkle of grated par-mesan
1 small tortilla	Egg white	1/2 cup black beans	Slices of avocado	Salsa and/or hot sauce

MOMS ON THE MOVE

A SAMPLE WORKOUT GUIDE FROM THE HEALTHY MOM ZONE STUDY

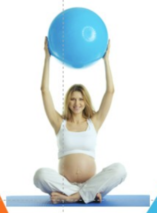

IRB# CATS00003752
PENNSTATE

Healthy Mom ZONE

2nd Trimester

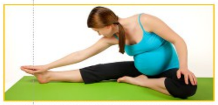

Workout Calendar

Workout	Sunday	Monday	Tuesday	Wednesday	Thursday	Friday	Saturday
Warmup							
Strength Workout							
Cardio Workout							

2nd Trimester - Strength

- **Braced Squat (2 Sets, 12-15 Reps)**
  - **(a)** Hold a weight plate in front of your chest with both hands, your arms completely straight
  - **(b)** Lower your body as far as you can
  - **(b)** Push your hips back and bend your knees
  - Pause then slowly push yourself back to starting position
  - You can modify the squats by placing a fitness ball behind your back and against the wall

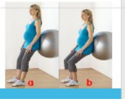

Supplement: Multimedia Appendix 1 [file resprot_v7i6e150_app1.pdf]
