# Supplementary material for: Individually Tailored, Adaptive Intervention to Manage Gestational Weight Gain: Protocol for a Randomized Controlled Trial in Women With Overweight and Obesity
Source: JMIR Res Protoc. 2018 Jun 8;7(6):e150. doi: 10.2196/resprot.9220 (PMC6015270; doi:10.2196/resprot.9220)
Supplement: Multimedia Appendix 2 [file resprot_v7i6e150_app2.pdf]

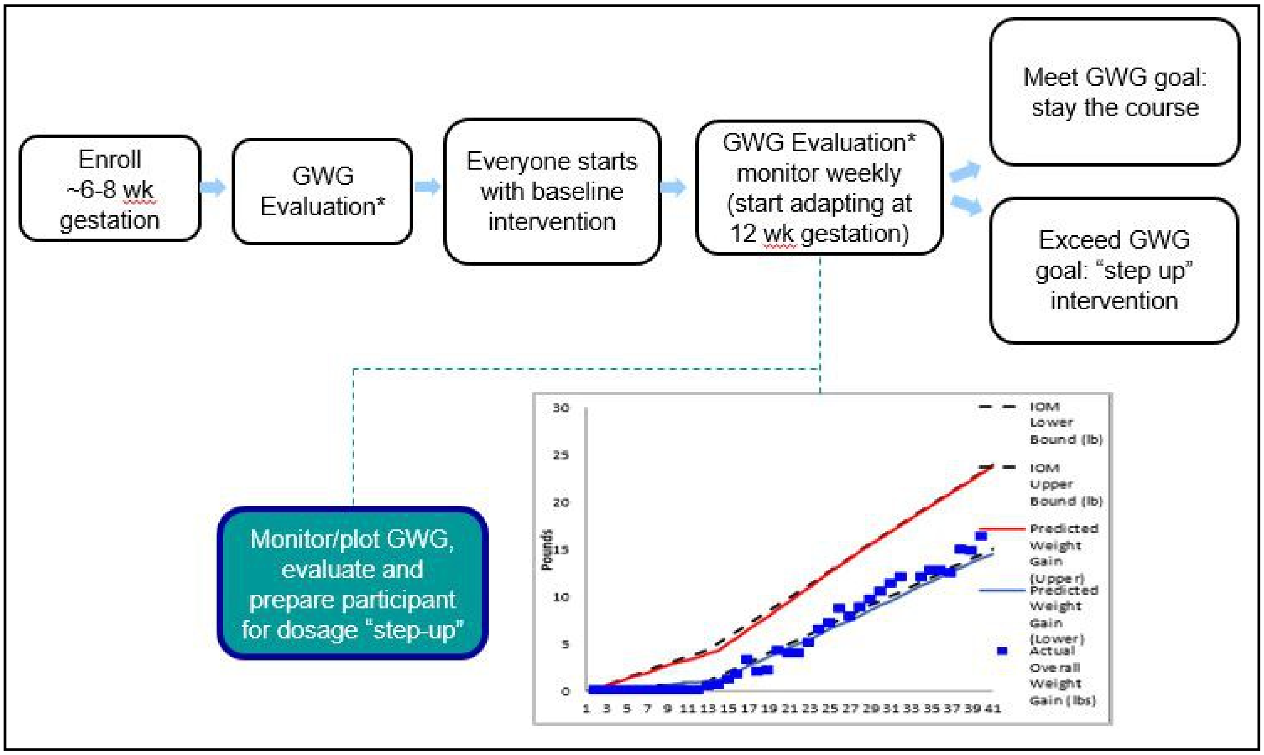

Note. Evaluation of a participant's measured weekly gestational weight gain (GWG) is plotted and compared against the Institute of Medicine (IOM)-determined amount of suggested weight gain based on her pre-pregnancy weight status [1], and decisions to adapt the intervention are made based on whether the participants is meeting or exceeding the goal (i.e., weight within the lower and upper IOM weight range).
