# Supplementary material for: Individually Tailored, Adaptive Intervention to Manage Gestational Weight Gain: Protocol for a Randomized Controlled Trial in Women With Overweight and Obesity
Source: JMIR Res Protoc. 2018 Jun 8;7(6):e150. doi: 10.2196/resprot.9220 (PMC6015270; doi:10.2196/resprot.9220)
Supplement: Multimedia Appendix 3 [file resprot_v7i6e150_app3.pdf]

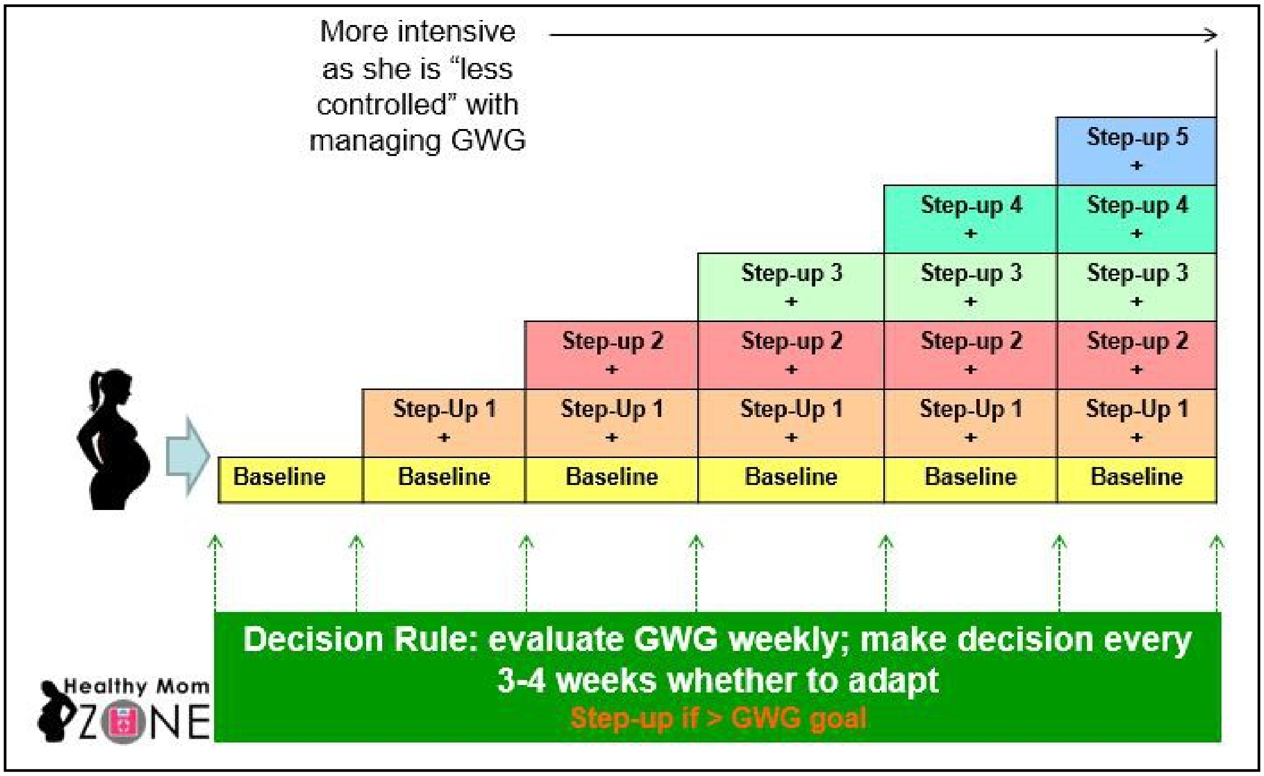

Note. GWG = gestational weight gain. The intervention dosage is adapted (e.g., "stepped up" or intensity of the intervention is increased) based on the extent to which a woman is meeting or not meeting her weight gain goals (determined by the decision rule process in Figure 6). A participant who is efficiently managing her weight may remain in the baseline intervention throughout the study duration whereas a participant who is having difficulty with managing her weight will receive additional intervention content and components (e.g., additional active learning sessions; meal replacements, etc.) to help bring her within her weight goal range [1].
